# Conductive Cotton by In Situ Laccase-Polymerization of Aniline

**DOI:** 10.3390/polym10091023

**Published:** 2018-09-14

**Authors:** Jing Su, Euijin Shim, Jennifer Noro, Jiajia Fu, Qiang Wang, Hye Rim Kim, Carla Silva, Artur Cavaco-Paulo

**Affiliations:** 1 International Joint Research Laboratory for Textile and Fiber Bioprocesses, Jiangnan University, Wuxi 214122, China; jingsu@ceb.uminho.pt (J.S.); kathyfjj@126.com (J.F.); qiang_wang@163.com (Q.W.); 2 Centre of Biological Engineering, University of Minho, Campus of Gualtar, 4710-057 Braga, Portugal; jennifer.noro@ceb.uminho.pt; 3 Department of Clothing and Textiles, Sookmyung Women’s University, Seoul 04310, Korea; euijin@sookmyung.ac.kr (E.S.); khyerim@sookmyung.ac.kr (H.R.K.)

**Keywords:** coatings, polyaniline, laccase, conductive cotton, 1-hydroxybenzotriazol (HBT)

## Abstract

Conductive cotton fabrics were obtained via in situ aniline polymerization by laccase from *Myceliophthora thermophila* under mild reaction conditions without the addition of strong proton acids. The reactions were conducted using two types of reactors, namely a water bath (WB) and an ultrasonic bath (US), and the role of a mediator, 1-hydroxybenzotriazol (HBT), on the laccase-assisted polymerization of aniline was investigated. A similar polymerization degree was obtained when using both reactors—however, the ultrasonic bath allowed the experiments to be conducted in shorter periods of time (24 h for WB vs. 2 h for US). The data obtained also revealed that the mediator (1-hydroxybenzotriazol-HBT) played a crucial role in aniline oxidation. A higher conversion yield and polymerization degree were obtained when the reaction was conducted in the presence of this compound, as confirmed by MALDI-TOF analysis. The cotton fabrics coated with polyaniline presented deep coloration and conductivity, especially when the mediator was included on the reactional system. The results obtained are a step forward in the enzymatic polymerization of aniline with the purpose of obtaining coloured conductive textile surfaces, with potential applications in wearable electronics.

## 1. Introduction

**Scheme 1:**
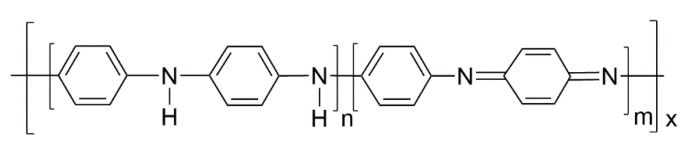
Three idealized oxidation states of polyaniline: Leucomeraldine, with n = 1, m = 0 (fully reduced state); Pernigraniline, with n = 0, m = 1 (fully oxidized state); the emeraldine n = 0.5, m = 0.5 (often referred as emeraldine base) [1,13].

Conducting polymers have been investigated for their potential use in many practical applications, such as energy-storage devices, anticorrosion protection, antistatic coating, biosensors, and others [1,2]. Among these polymers, polyaniline (PANI) is considered one of the most versatile conducting polymers due to its high electronic conductivity and excellent chemical stability [3]. PANI was first synthesized by Letheby during electrolysis of aniline sulfate [4], and since then, many researchers started to explore its potential applicability. The synthesis of PANI can be achieved via different methods, namely with oxidant (e.g., chemical synthesis, interfacial polymerization, etc.) or without oxidant (e.g., electrochemical synthesis) [2,5,6,7]. Among these methods, a new classification was made based on the application of a template during synthesis, namely the solid template approach, the molecular template approach, and the template-free approach [8]. For the solid template approach, a template membrane is usually required to provide control over the size and shape of the nanostructures of the polymer. However, the template needs to be removed and may disorder the major nanostructures formed. The molecular template involving the use of surfactants, surface micelles, and liquid crystalline phases has been developed as an easy alternative to the solid template without further separation [9]. The template-free approach has also been studied to circumvent the drawbacks of the template approach, such as high cost and property damage, which is now highly attractive due to its easy operation and low cost. Although the formation of the homopolymer PANI could be achieved in multiple pathways, the conjugation mechanism of polyaniline is unique due to a combination of benzenoid and quinoid rings leading to three different oxidation states in different conditions (Scheme 1) [1,10]. Studies have shown that most polyanilines are one of these three states, or physical mixtures of these components. The emeraldine base form of PANI can be doped by a sufficiently strong proton acid like H_2_SO_4_, HNO_3_, HCl, and others to achieve high conductivities [11,12].

Traditional chemical and electrochemical methods for polyaniline production generally involve harsh conditions which are harmful to the environment [10]. Recently, enzymatic catalysis has been considered as an attractive alternative for the green synthesis of conductive PANI, since the reactions can be performed under mild conditions and are kinetically controllable. To date, only few studies have reported the use of enzymes for the oxidation of aniline [2,10,13,14,15,16]. Horseradish peroxidase (HRP) and laccases have been explored to obtain PANI. Liu et al. synthesized a water-soluble and conducting polyaniline (PANI)/sulfonated polystyrene (SPS) complex using HRP as a catalyst. The resulting form of electroactive PANI was similar to the one obtained chemically, but was more environmentally compatible [15]. Sahoo et al. also performed a HRP-catalyzed enzymatic polymerization of PANI with polyethylene glycol(PEG)-hematin in the presence of different templates, like poly(vinylphosphonic acid) (PVP) and poly(4-styerenesulfonate) (SPS) [17]. Compared with peroxidases [16,18], laccases are more environmentally friendly since the catalysis reactions are carried out without the addition of diluted hydrogen peroxide. Laccases catalyse the oxidation of various substrates such as phenolics and aromatic amines, which perform the four-electron reduction of dioxygen to water [19]. For polyaniline, the formation of a polymer chain starts with the oxidation of aniline monomer by laccase, as well as dimers and oligomers [20]. Afterwards, the polymerization proceeds by non-coupling of the oxidized products, during which the abstraction of hydrogen atoms are produced as new covalent bonds between aniline molecules for the chain polymer structure, and controlled reaction conditions are needed to avoid resulting side-effects [3].

Laccases from different sources have therefore been investigated as catalysts for the synthesis of polyaniline. Streltsov et al. presented a method to obtain electroconductive PANI on micelles of dodecylbenzenesulfonic acid sodium salt (DBSNa) using laccase from *Trametes hirsuta* as a biocatalyst [21]. This enzymatic synthesis produced a stable dispersion of DBSNa-polyaniline nanoparticles, which the corresponding product, performing at pH 4.0, presented the spectrum characteristic of the electroconductive polyaniline. Zhang et al. [13] performed the polymerization of aniline with laccase from *Aspergillus* in a water dispersion of the SDBS system, and immobilized this SDBS-doped PANI onto a cotton surface, which greatly improved the electrical properties of the cotton, such as the antistatic property and electromagnetic shielding effect. Vasil’eva et al. [22] developed a one-step polymerization method to obtain in situ optically active polyaniline, deposited on the surface of glass slides using laccases from *Trametes hirsuta*. The polymerization was conducted under aerobic conditions in weak acidic solution and the optical activity of polyaniline was confirmed.

**Scheme 2:**
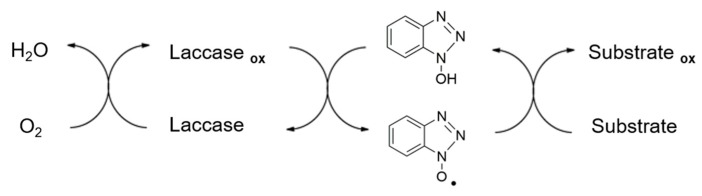
The role of the 1-hydroxybenzotriazol (HBT) mediator on the oxidation reactions mediated by laccase (adapted from [26]).

During synthesis, experimental factors like reactors, temperature, and pH environment could potentially affect polymerization. Moreover, the properties of PANI are strongly influenced by the different templates or additives applied in the reaction system. In some studies, 1-hydroxybenzotriazole (HBT) was considered as a diffusible radical mediator during the oxidation of substrates like phenols and aromatic amines, and its presence induced a positive effect on the reaction conversion yields [23,24,25,26] (Scheme 2).

Laccases are able to catalyse the oxidation of a broad range of phenolic and non-phenolic substrates with the concomitant reduction of oxygen to water, rather than chemical or peroxidase-based oxidative polymerizations. On this account, different forms of laccase catalysts (PEGylated and immobilized) were previously prepared and applied to perform the polymerization of catechol and *p*-phenylenediamine, using different fabrics as enzyme containers (PET, cotton, and wool) [26,27,28]. Poly(catechol) and poly(*p*-phenylenediamine) obtained after in situ enzymatic polymerization were able to confer colour and antimicrobial character to the coated fabrics. Due to its unique physical and chemical properties such as good flexibility, high porosity, and breathability, cotton fabric has been considered as one of the most commonly used biodegradable materials for wearable textiles and personal care products. Moreover, the active hydroxyl groups of cotton fabric ensure easy modification and good distribution of functional materials on the fabric [29,30,31].

In this study, our goal has been to develop conductive cotton fabrics by in situ laccase-polymerization of aniline using a template-free approach in mild conditions without strong proton acid. Two different reactors were used to conduct the experiments, namely a water bath and an ultrasonic bath. The effect of the addition of a mediator on the reactional system, namely 1-hydroxybenzotriazole (HBT), was assessed. The polymerization was followed in a solution by UV-Vis spectroscopy, and the polymers obtained were characterized by ^1^H NMR and MALDI-TOF spectroscopy. The coated fabrics were evaluated by spectral values determination (checksum K/S) and electric conductivity evaluation. We envisaged using the natural textile surfaces to develop textile conductive devices with potential applications, namely on wearables.

## 2. Experimental Section

### 2.1. Materials

Laccase (EC 1.10.3.2.) from *Myceliophthora thermophila* (Novozymes A/S 51003, batch number: OMN07012) was supplied by Novozymes, Frederiksberg, Denmark. Aniline and 1-hydroxybenzotriazole (HBT) were purchased from Sigma Aldrich, Madrid, Spain. Deuterated chloroform was obtained from Cortecnet, Voisins-Le-Bretonneux, France.

### 2.2. Experiments

#### 2.2.1. Evaluation of Enzyme Activity and Stability

The effect of a water bath and ultrasonic bath on the activity and stability of native laccase was evaluated. For this, the enzyme was incubated under the same conditions used for aniline polymerization: 25 U/mL laccase was incubated with acetate buffer (pH 4) at 25 °C in a water bath for 24 h and in an ultrasonic bath for 2 h. Aliquots of enzyme solution were taken at different periods of incubation, and the activity of laccase was measured against ABTS according to the methodology described by Childs and Bardsley [32].

The enzyme-diluted solution was mixed (1:1) with ABTS (5 mM) in acetate buffer and the increase in absorbance was followed at 420 nm every minute until 10 min of incubation. The spectrophotometer was zeroed with the ABTS zero sample, which contained a mixture of acetate buffer (0.1 M, pH = 4) and ABTS solution. The experiment was performed at 25 °C. The activity in units (U) was defined as the amount of enzyme required to oxidize 1 µmol of ABTS per minute and was calculated by following Equation (1) [33]:

(1) $$\mathrm{Enzyme}\;\mathrm{activity}=\frac{\Delta \mathrm{OD}\times V_{total}}{\varepsilon_{ABTS}\times d\times V_{enzyme}}\times N$$

$V_{total}:$ total volume of test solution; $V_{enzyme}:$ total volume of enzyme solution; $\varepsilon_{ABTS}$: 36,000 L/(mol·cm); *d*: light pass length (cm); *N*: enzyme dilute times.

#### 2.2.2. In Situ Laccase Polymerization of Aniline on Cotton

The aniline was oxidized in situ into polyaniline by native laccase in acetate buffer (0.1 M; pH 4) using two different devices, namely water bath and ultrasonic (USC600TH, VWR International Ltd., Radnor, PA, USA; frequency 45 kHz and power of 120 W) in the absence and presence of a HBT mediator. Thus, aniline (50 mM) was incubated with laccase (25 U/mL) at 25 °C under different conditions: (a) without HBT (10 mM) in a water bath for 24 h; (b) with HBT in a water bath for 24 h; (c) without HBT in an ultrasonic bath for 2 h; (d) with HBT in an ultrasonic bath for 2 h. For all the experiments, a cotton fabric (2 × 2 cm) was placed in the reaction system to proceed the in-situ polymerization and coating. After the reaction, the cotton samples were vigorously washed with distilled water to remove the by-products and remaining starting reagents. Afterwards, the coated cotton fabrics were placed in a convection oven at 25 °C until totally dried. The final precipitates were washed and centrifuged several times with water and freeze-dried for posterior analysis.

#### 2.2.3. UV-Visible-NIR (Ultraviolet–Visible-Near-Infrared) Spectroscopy Evaluation

The polymerization of aniline by laccase, in the presence and absence of the HBT mediator, was followed by UV-Visible-NIR spectroscopy using a UV-3101PC spectroscope (Shimadzu, Kyoto, Japan).

#### 2.2.4. ^1^H NMR Spectra

The precipitates obtained after washing and centrifugation were dissolved in deuterated chloroform for ^1^H NMR evaluation. The spectra were acquired in a Bruker Avance III 400 (400 MHz) (Bruker, Bremen, Germany) using the peak solvent as an internal reference.

#### 2.2.5. MALDI-TOF (Matrix-Assisted Laser Desorption/Ionization with Time-of-Flight) Spectroscopy Analysis

The polymers were analysed by Matrix-Assisted Laser Desorption/Ionization with time-of-flight (MALDI-TOF) using 2,5-dihydroxy benzoic acid (DHB) as the matrix (≥99.5%). The mass spectra were acquired on an Ultra-flex MALDI-TOF mass spectrophotometer (Bruker Daltonics GmbH, Bremen, Germany) equipped with a 337 nm nitrogen laser. For this, the samples were dissolved in a TA30 (30% acetonitrile/70% trifluoracetic acid) solution and mixed with a 20 mg/mL solution of DHB (1:1). Then a volume of 2 μL was placed in the ground steel plate (Bruker part n 209519) until dry. The mass spectra were acquired in linear positive mode.

#### 2.2.6. Scanning Electron Microscopy

The surface topography and chemical composition of the coated samples were investigated by SEM/EDS evaluation. All the fabric samples were added to aluminium pin stubs with electrically conductive carbon adhesive tape (PELCO Tabs™), with the excess removed using compressed air. Samples were coated with 2 nm of Au for improved conductivity. The aluminium pin stub was then placed inside a Phenom Standard Sample Holder, and different points for each sample were analysed for elemental composition. The samples were characterized using a desktop scanning electron microscope (SEM) coupled with energy-dispersive X-ray spectroscopy (EDS) analysis (Phenom ProX with an EDS detector (Phenom-World BV, Eindhoven, The Netherlands)). All results were acquired using the ProSuite software integrated with Phenom Element Identification software (Thermofisher Scientific, Eindhoven, The Netherlands), which allowed the quantification of the elements concentration present in the samples, expressed either in weight or atomic concentration.

#### 2.2.7. Conductivity of Coated Cotton Fabric

Electric conductivity was measured on a Fluke 123 Scopemeter (20 Mhz) (Fluke, Eindhoven, The Netherlands) using a two-point probe technique by placing them under a pre-defined distance between two spots of the fabric samples. The conductivity was calculated according to the following Equation (2):

(2) $$\mathrm{Conductivity}\;\left(\sigma \right)=\frac{1}{\rho }\left({S\cdot \mathrm{cm}}^{-1}\right)$$

obtained from the calculation *R_O_* = *ρ* (*L*/*A*), where *R_O_*: resistance in ohm.cm; *ρ*: resistivity (cm); *L*: distance between electrodes (6 mm); *A*: area of material section (6 × 0.5 mm).

#### 2.2.8. K/S Evaluation of Coated Cotton Fabrics

The colour strength (checksum *K/S*) was evaluated by using a Datacolor apparatus (Datacolor, NJ, USA) at standard illuminant D65 with the Kubelka-Munk equation, Equation (3), in which *K* is the absorbance coefficient, *S* is the scattering coefficient, and *R* is the reflectance ratio.

Since not all the samples presented the same maximum wavelength, the data is presented as the sum of all *K/S* values obtained at the wavelength range of 400–700 nm (checksum *K/S*). The measurements were done in triplicate, and the data presented are the mean values of these measurements.

(3) $$\frac{K}{S}=\frac{{\left(1-R\right)}^{2}}{2R}$$

## 3. Results and Discussion

### 3.1. Evaluation of Laccase Activity and Stability

**Figure 1:**
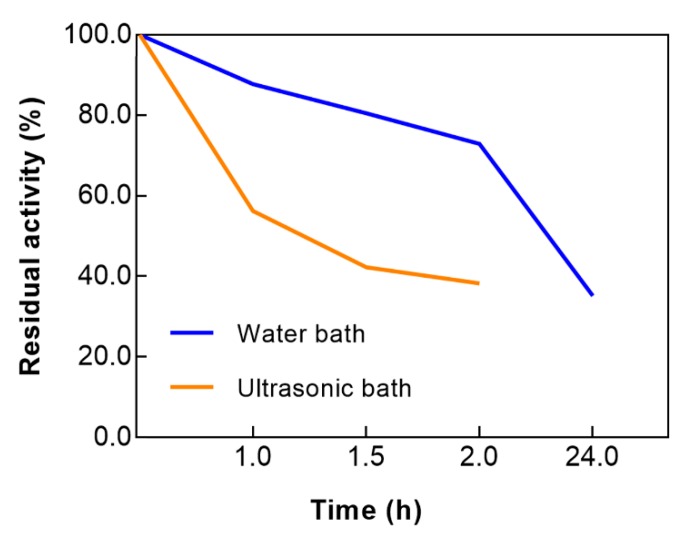
Residual enzyme activity of laccase incubated in a water bath (blue line) and in an ultrasonic bath (orange line) in acetate buffer, pH 4 at 25 °C.

Generally, proteins are sensitive to harsh conditions like high temperature and extreme pH levels. However, high-energy environments like ultrasound are also thought to have a negative effect on enzyme activity and stability under certain conditions [33,34,35]. An enzyme’s tolerance towards ultrasound might depend on the operational parameters such as power rating and ultrasound frequency. To predict the effect of high-energy environments (ultrasound) on laccase stability, the activity of laccase was evaluated during processing. Figure 1 shows the residual activity of laccase when incubated using a water bath and an ultrasonic bath reactor. The results obtained show that after incubation using acetate buffer (pH 4) in the water bath for 24 h, the enzyme retained almost 35.2% of its initial activity. In the first two hours of incubation using the water bath, the enzyme lost only 30% of its initial activity. When the ultrasonic bath was applied, the half-life of the enzyme was greatly reduced in the first 2 h. These results are in accordance with our previous findings related with the polymerization of catechol under high-energy environments [28]. We have previously found that since polymerization occurs mostly in the first hour of incubation, the activity loss will not restrict the final amount of polymer produced, nor the coating efficiency.

### 3.2. In Situ Polymerization of Polyaniline and Characterization

#### 3.2.1. UV-Vis-NIR Spectroscopy Observation

**Figure 2:**
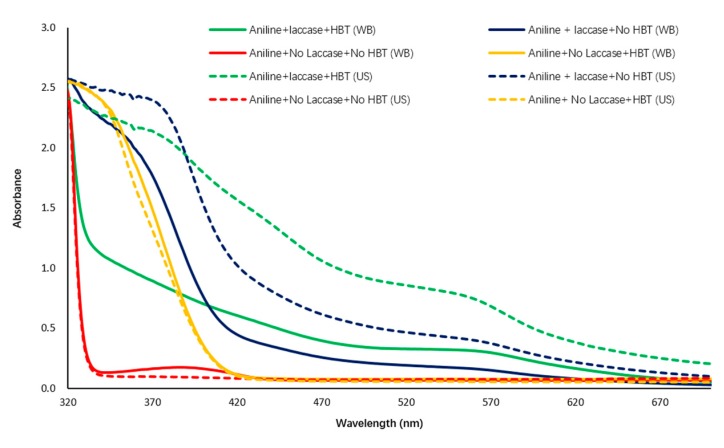
UV-Vis-NIR (Ultraviolet–visible-near-infrared) spectra of polyaniline solutions. Experiment conditions: 50 mM aniline incubated with 25 U/mL laccase at 25 °C using: (a) HBT (10 mM) in a water bath for 24 h; (b) without HBT in a water bath for 24 h; (c) HBT (10 mM) in an ultrasonic bath for 2 h; (d) without HBT in an ultrasonic bath for 2 h.

Figure 2 shows the effect of mediator HBT on the UV-Vis-NIR absorption spectra of aniline solutions after polymerization by laccase using the water bath and the ultrasonic bath reactors. The maximum absorption wavelength shifted from 280 to 380 nm and increased in intensity when laccase was applied together with HBT, indicating aniline polymerization and alignment of the polymer structure [13]. Moreover, a new peak at around 570 nm can be detected for samples incubated in the presence of the HBT mediator, for both water bath and ultrasonic bath reactors. This new peak is related with the new green-yellowish coloration of the solutions. From the spectra obtained, the positive role of the mediator on the oxidative polymerization of aniline is also perceptible. A higher level of absorbance of the final solutions was detected for samples incubated in the presence of the HBT mediator, independently on the reactor used to conduct the experiments. This corresponds to higher reaction rates and higher polymerization degrees, as will be discussed further. Contrarily to previous reports where the degree of conjugation and conductivity of the polyaniline declined with an increase in temperature, our samples behaved differently. The polymerization of aniline reached similar values when conducted at 4 °C (data not shown) and 25 °C, confirming consistency with the optimum temperature of the enzyme (50 °C) and showing a potential easy method of polymerization. Several factors are known to influence the polymerization rates, namely the size of the reaction flask, stirrer speed, and the reaction temperature [1]. Another determinant factor for aniline polymerization is the pH. Several works report that the optimum pH for obtaining a high degree of conjugation and conductivity is between the range of 3.0 and 4.5 [13,20]. Herein, pH 4.0 has proved to have a positive effect on producing conductive polyaniline, as also confirmed by others [1]. The pH level applied might have negatively influenced the enzyme stability during processing, as observed previously in Figure 1.

#### 3.2.2. ^1^H NMR Spectroscopy

The final polymers were analysed by ^1^H NMR. After analysis it was possible to detect the absence of the OH peak and the change in the chemical shifts of the aromatic protons of HBT, after the polymerization reaction (data not shown). This result is consistent with our findings about the crucial role of HBT on the enzymatic polymerization of aniline.

#### 3.2.3. MALDI-TOF Mass Spectrometry

**Figure 3:**
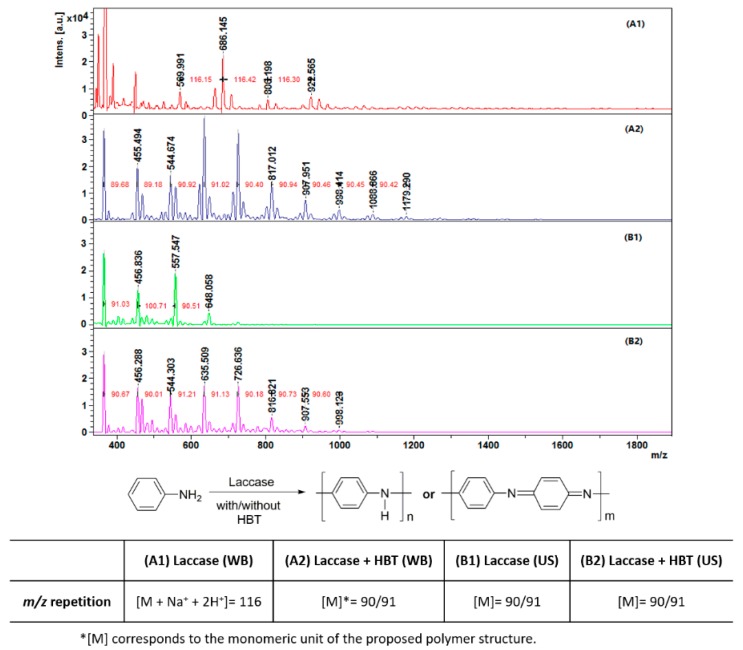
MALDI-TOF (Matrix-Assisted Laser Desorption/Ionization with time-of-flight) of polyaniline obtained by using: (**A1**) Aniline + laccase + No HBT-water bath for 24 h; (**A2**) Aniline + laccase + HBT-water bath for 24 h; (**B1**) Aniline + laccase + No HBT-ultrasonic bath for 2 h; (**B2**) Aniline + laccase + HBT-ultrasonic bath for 2 h.

MALDI-TOF data in Figure 3 also confirmed the role of the HBT mediator on the polymerization of aniline using both a water bath and ultrasonic bath. From the results, it can be seen that longer oligomers/polymers were obtained in the presence of HBT, and short oligomers were obtained in its absence. From literature, N-OH-type mediators such as HBT are likely to act through a radical hydrogen atom transfer (HAT) route. During the laccase oxidation reaction, the HBT mediator could form cation radicals by abstracting a proton from the substrate and converting it into a radical, which can oxidize the substrate having a higher redox potential than the mediator itself, and thus favour enzymatic polymerization [25,36,37]. From our data, the role of HBT on the radical polymerization of aniline was confirmed, although the detailed pathways are not explained.

Table 1 presents the characterization of the oligomers/polymers obtained after the oxidation reaction. The degree of polymerization (DP) and the *M*n, *M*w values were calculated based on MALDI-TOF spectra analysis. The results revealed that the polymerization conducted using both water bath and ultrasonic bath reactors gave rise to oligomers/polymers with similar DP. However, it is noteworthy that the ultrasonic bath allowed for a shorter reaction time with reduced costs in terms of energy consumption. The mass transport phenomena and associated higher substrate accessibility were the most critical factors deciding the results’ similarity [28,38]. As previously mentioned, the HBT mediator had a similar effect when using both reactors, allowing an increase in the DP from 5.5 to 7.5 and 7.0 for the water bath and ultrasonic bath, respectively.

### 3.3. Characterization of the Coated Fabrics

#### 3.3.1. Colour Evaluation of Polyaniline Coated Fabrics

**Figure 4:**
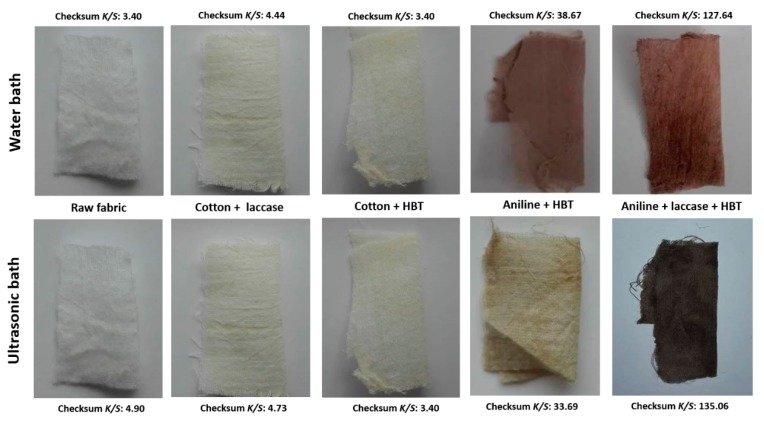
Photographs and spectral values of samples coated using different reactors, water bath and ultrasonic bath, under different conditions: raw fabric; cotton + laccase; cotton + HBT; cotton+ laccase + no HBT; cotton + laccase + HBT.

Many polymers synthesized by enzymes can be described as potential colorants of textile fibres, as previously explored in literature [39,40,41]. In this work, polyaniline also endowed a light greenish-brown colour to the cotton fabrics. Visually, there is a considerable difference between samples coated in the presence and absence of HBT (Figure 4). This difference was precisely quantified by spectra evaluation (checksum *K/S*). The data reveals higher levels of coating in the presence of the HBT mediator, corresponding to longer polymers and higher amounts of oligomers/polymers produced. This led to the deposition of a higher amount of colorant at the surface of the cotton fabrics. The difference in the color of the cotton fabrics is clearly visible with the naked eye, where the originally white cotton has become dark green after coating with polyaniline, especially when the experiment was conducted in the ultrasonic bath. Incubation in the water bath gave rise to samples heterogeneously coated with brown-green coloration.

As it is known, the oxidation of aniline at different pH conditions produces polyaniline with different characteristics. The most widely used synthetic route for conducting PANI is in acidic aqueous media by forming a polymer with a green colour, which is conductive. When in an alkali environment, polyaniline presents a blue colour and is non-conducting. The third type is the yellowish PANI, which can be obtained in mildly acid conditions [3]. Our study was conducted at pH = 4, and as mentioned by others [13], this pH allowed for the production of a conductive polyaniline with a high degree of conjugation, indicating that laccase from *Myceliophthora thermophila* catalyzed the polymerization of aniline in the presence of the HBT mediator to form water-soluble conductive polyaniline under milder conditions than those of traditional chemical and enzymatic catalysis methods previously reported.

#### 3.3.2. Scanning Electron Microscopy and Energy-Dispersive X-ray Spectroscopy

**Figure 5:**
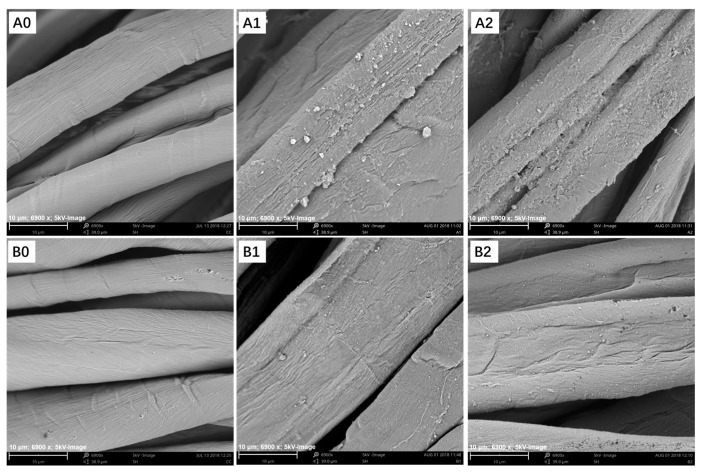
Scanning electron microscope (SEM) images of cotton fabrics coated with polyaniline: (**A0**) cotton fabric control-water bath; (**A1**) fabric coated with polyaniline-water bath (no HBT); (**A2**) fabric coated with polyaniline-water bath (with HBT); (**B0**) cotton fabric control-ultrasonic bath; (**B1**) fabric coated with polyaniline-ultrasonic bath (no HBT); (**B2**) fabric coated with polyaniline-ultrasonic bath (with HBT).

As shown in Figure 5, the morphology of the coated conductive cotton was investigated using SEM microscopy. After in situ polymerization of aniline, many granules of doped polyaniline were deposited on the cotton surface, enhancing its electrical properties. The coating resulted from the hydrogen bonds between the polyaniline and the hydroxyl groups of cotton. The protonation of the polyaniline imine nitrogen through interactions with the hydroxyl groups of the cotton fibers reinforced the interactions between the polyaniline chains and cotton [13].

The EDS analysis also confirmed the deposition of polyaniline onto cotton surfaces by identification and quantification of the elements present in the cotton samples after coating, expressed by both weight and atomic concentration. Different spots on the samples were selected for the quantification, and the values obtained were very similar. From Table 2 it can be seen that the results for the control cotton confirmed the presence of carbon and oxygen and the absence of nitrogen. After coating, the nitrogen element was detected in all the cotton samples, especially on samples coated in the presence of the HBT mediator, in both water bath and ultrasonic bath reactors, confirming the presence of polyaniline after coating.

#### 3.3.3. Electric Conductivity of Fabrics Coated with Polyaniline

Different substrates have been coated with polyaniline, like silica, glass, quartz, polyester, wool, and cotton, to achieve conductivity [42,43,44,45,46]. In these works, conducting polymers are deposited on the fabric’s surface, chemically or electrochemically, with the need of extra chemical reagents. Considering these drawbacks, a green polymerization, assisted by laccase, was used herein to confer electrical properties to the cotton fabrics.

**Figure 6:**
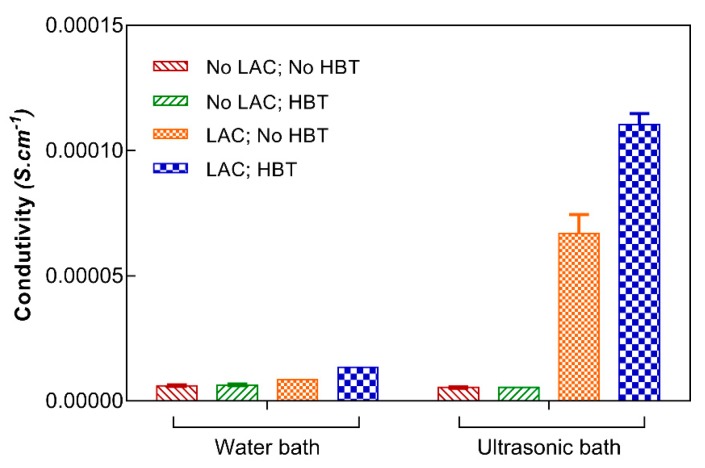
Conductivity of cotton fabrics after coating with polyaniline.

During the in situ reaction, two events can occur: the deposition of polyaniline onto the textile substrate and/or the precipitation of its homopolymer in the reaction solution. The electrical conductivity of coated cotton samples is a function of the extent of polyaniline deposition on the fabric substrate, and this electrical conductivity was evaluated and presented in Figure 6. From the data obtained, one can observe that the samples coated with polyaniline assisted by laccase in the presence of the HBT mediator (blue colour) revealed higher conductive behaviour compared with the samples coated in the absence of HBT (orange colour). The differences in conductivity obtained when the different reactors were used is also noteworthy. The samples incubated in the water bath presented a negligible conductive character when compared with the samples incubated in the ultrasonic bath. As previously stated, the intrinsic phenomena involving processing with this device allowed a higher mass transfer from the bulk to the fabric surface, increasing coating levels, thus incrementing the conductivity of the fabrics.

## 4. Conclusions

Conductive cotton was prepared by in situ laccase-polymerization of aniline. Compared with the chemical methods generally used, this methodology allows the oxidation of aniline without the need of additives, using only the atmospheric oxygen as an oxidant.

The use of the ultrasonic bath as reactor device allowed for high conversion rates to be achieved in short periods of time. The HBT mediator also played an important role in aniline oxidation, leading to longer oligomers/polymers. The coated fabrics acquired a conductive character when oxidation took place at pH = 4, 25 °C.

This study has been one of few attempts on the oxidation of aniline without the use of additives. Despite the low conductivity results, it is likely to grant better understanding on how to apply coloured conductive polyaniline to textiles and turn them into conductive devices. Moreover, polyaniline production implied the use of a milder methodology than that usually applied for the production of this polymer.

## Figures and Tables

**Table 1:** Polyaniline characterization: *M*n, *M*w, Polydispersity (PDI), and average polymerization degree (PD) using a water bath and an ultrasonic bath reactors; the values were obtained by MALDI-TOF analysis.

	Water Bath Reactor (24 h)		Ultrasonic Bath Reactor (2 h)	
	Laccase	Laccase + HBT	Laccase	Laccase + HBT
*m/z*_(max)_	922	1179	648	998
DP	5.5	7.5	5.5	7.0
*M*w	507	675	511	626
*M*n	455	614	484	572
PDI	1.11	1.09	1.05	1.09

**Table 2:** Elements characterization (on different spots of the sample) on coated and non-coated fabrics, by energy-dispersive X-ray spectroscopy (EDS) analysis.

Element conc. (%)	Element	Control Fabric		Water Bath Reactor (24 h)				Ultrasonic Bath Reactor (2 h)			
				Laccase		Laccase + HBT		Laccase		Laccase + HBT	
		Spot 1	Spot 2	Spot 1	Spot 2	Spot 1	Spot 2	Spot 1	Spot 2	Spot 1	Spot 2
Atomic conc.	Carbon	80.23	78.53	59.67	59.47	66.83	74.11	74.43	79.63	64.05	67.62
	Oxygen	19.77	21.47	34.36	32.62	22.54	13.00	22.85	18.51	29.63	26.60
	Nitrogen	0	0	5.97	7.91	10.64	12.89	2.72	1.86	6.32	5.78
Weight conc.	Carbon	75.29	73.30	53.08	52.31	61.17	69.62	68.89	74.80	57.76	63.14
	Oxygen	24.71	26.70	40.72	39.58	27.48	16.26	28.17	23.16	35.59	30.90
	Nitrogen	0	0	6.20	8.11	11.35	14.12	2.94	2.04	6.65	5.96
